# Egr2 to the rescue: nanoparticles revitalize natural killer cells in the fight against cancer

**DOI:** 10.1038/s44318-024-00144-y

**Published:** 2024-06-17

**Authors:** Aline Pfefferle, Santosh Phuyal, Herman Netskar, Karl-Johan Malmberg

**Affiliations:** 1 Center for Infectious Medicine, Department of Medicine Huddinge, Karolinska Institutet, Stockholm, Sweden; 2 Precision Immunotherapy Alliance, University of Oslo, Oslo, Norway; 3 Department of Cancer Immunology, Institute for Cancer Research, Oslo University Hospital, Oslo, Norway

**Keywords:** Cancer, Immunology

## Abstract

A new study follows a nanoparticle-based gene silencing approach to overcome natural killer cell dysfunction in cancer.

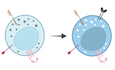

## Teaser

Reversal of tumor-induced natural killer (NK) cell dysfunction remains a major hurdle to overcome for the clinical success of immunotherapies based on NK cells targeting solid tumors. New research in The EMBO Journal identifies the transcription factor early growth response gene 2 (Egr2) and diacylglycerol kinase DGKα as two negative regulators of NK cell dysfunction (Sabag et al, 2024).

**Figure 1:**
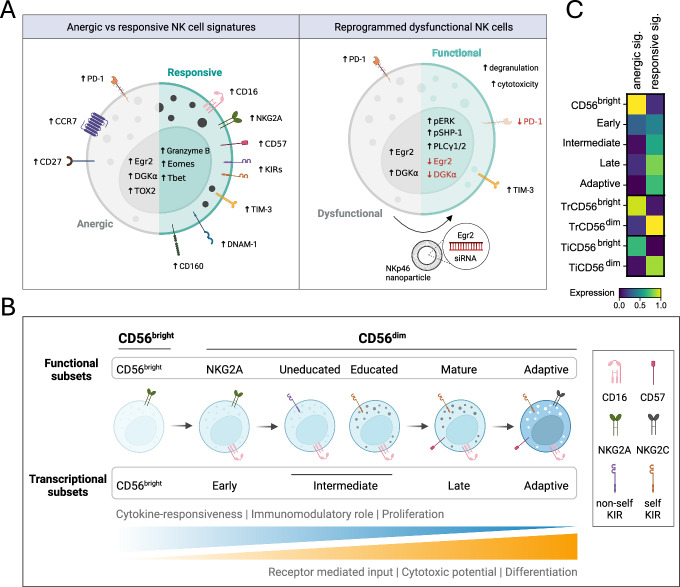
Anergic and responsive NK cells in relation to NK cell differentiation and education. (**A**) Graphical summary of the anergic and responsive gene signatures (left panel) and the functional reprogramming of anergic NK cells (right). (**B**) Overview of NK cell differentiation and education, at the functional and transcriptional level. (**C**) Scoring of the anergic and responsive gene signatures in a single-cell RNAseq dataset comprising of blood, tissue-resident (Tr), and tumor-infiltrating (Ti) NK cells. The scale represents gene set activity computed by AUCell.

The tumor microenvironment (TME) is a hostile place for NK cells, as hypoxia, nutrient deprivation, and suppressive immune cells can all negatively impact NK cell functionality. Hence, overcoming this TME-induced NK cell dysfunction is an attractive strategy to improve NK cell-based immunotherapies for solid tumors. This could be achieved either via targeting the elements of the TME that suppress NK cell function or via genetic engineering of NK cells. Sabag et al, (2024) tackled NK cell dysfunction from a basic NK cell point of view, by first deciphering NK cell ‘anergy’, a different type of dysfunction essential to maintaining tolerance against self (Fig. 1A). They identified the zinc finger transcription factor Egr2 and diacylglycerol kinase DGKα as critical regulators of NK cell anergy, with increased expression observed in both ‘anergic’ and ‘exhausted’ NK cells from a xenogeneic tumor-grafted mouse model. Notably, Egr2-mediated DGKα expression was previously reported to underlie the anergic phenotype in T cells, highlighting the important role of these genes in regulating functionality across multiple cytotoxic lymphocytes (Zheng et al, 2012).

By silencing Egr2 and DGKα, ζ, the authors managed to enhance functionality of ‘anergic’ NK cells as observed by increased degranulation, ERK phosphorylation, and restoration of intracellular calcium. Silencing of Egr2 in the dysfunctional NK cells also led to a decreased expression of the immune checkpoint protein PD-1. Furthermore, the authors found that high Egr2 and DGKα expression in NK cells infiltrating acute myeloid leukemia and glioma tumors was associated with significantly reduced patient survival compared to NK cells exhibiting low Egr2 and DGKα expression profiles. Taken together, these findings underscore the potential of modulating intracellular signaling pathways to overcome immune cell dysfunction in cancer and viral infections. One innovative aspect of their study lies in the use of an anti-NKp46-labeled nanoparticle-based delivery system to target Egr2 in NK cells in an organotypic spheroid model and a mouse model of pancreatic cancer (Fig. 1A). This approach minimized off-target effects, as Egr2 expression is not restricted to NK cells. In both models, reversal of the NK cell dysfunctional state attenuated tumor volume and growth rate. Functional ex vivo analyses showed high degranulation in NK cells derived from mice that received Egr2 siRNA encapsulated nanoparticles directly linking NK cell activity to reduced tumor growth. These observations demonstrate the usability and efficacy of nanoparticles in modulating NK cell activity in situ. Whether this approach gains momentum for harnessing the full potential of nanoparticle-based platforms for gene manipulation in immune cells remains to be seen. One strategy could perhaps be labeling nanoparticles with a combination of targeting receptors to increase on-target delivery. Anergic NK cells treated with Egr2 siRNA exhibited increased cytotoxic potential as evidenced by improved tumor control, indicating a reversal of the dysfunctional state. By restoring the functional capabilities of NK cells, this approach holds promise for developing more effective NK cell-based immunotherapies.

While these results are encouraging, we would like to emphasize here the value of their interpretation in the broader context of NK cell biology, particularly NK cell education and differentiation (Pfefferle et al, 2020) (Fig. 1B). The authors’ initial separation of peripheral blood NK cells into ‘anergic’ and ‘responsive’ populations was based on the complete absence or expression of at least one inhibitory receptor, namely NKG2A, KIR2D, and KIR3DL1. In our view, this strategy does not distinguish between the functional effects of NK cell differentiation and education, which together define the functional potential of the cell. Differentiation of NK cells, the transition of immunomodulatory CD56^bright^ NK cells into terminally differentiated CD56^dim^ NK cells, is associated with increased cytotoxic potential, whereby intermediate phenotypes are defined by the expression of surface markers including NKG2A, KIRs, CD57, and CD16 (Björkström et al, 2010). The CD56^bright^ NK cells are highly responsive to cytokine stimulation and poorly cytotoxic despite expressing the inhibitory receptor NKG2A. These cells are transcriptionally distinct from CD56^dim^ NK cells; dominant transcriptional factors of CD56^bright^ cells include TCF1, LEF, and MYC, while CD56^dim^ cells highly express PRDM1, MAF, and TBX21 (Collins et al, 2019).

Importantly, the functional fine-tuning of the CD56^dim^ population through the process of education is distinct from differentiation (Anfossi et al, 2006). Differentiation provides a functional template, which is tuned by receptor-ligand interactions to self HLA class I molecules during education. Expression of a specific KIR only results in increased cytotoxic potential if the corresponding ligand is present (Horowitz et al, 2016). This is a dynamic process as transfer into a new HLA environment redefines the educated and uneducated populations (Ebihara et al, 2013). Natural killer cell education and subsequent effector functions have been associated with several phenotypic changes, including modulation of activating (DNAM-1) and inhibitory receptors (self-specific KIR) at the cell surface, altered signaling through SHP-1, mTOR signaling, and structural remodeling of secretory lysosomes (Goodridge et al, 2019; Marçais et al, 2017; Wu et al, 2021; Netskar et al, 2023). Strikingly, despite these distinct phenotypes, previous attempts to define a clear gene signature for NK cell education have largely failed (Goodridge et al, 2019; Holmes et al, 2021). Therefore, we suggest that the strong transcriptional signature identified by Sabag et al, (2024) is likely due to the comparison between receptor-negative and receptor-positive NK cells, representing different stages of differentiation rather than hyporesponsive versus functional educated states.

Some of the significantly downregulated genes the authors detect in ‘anergic’ NK cells are typically expressed by CD56^dim^ cells, such as FCGR3A (CD16), B3GAT1, GZMB, CD226 (DNAM-1), CD160, and TBX21, while the increased expression of CCR7 and CD27 correlates with CD56^bright^ cells (Collins et al, 2019). Scoring for the ‘anergic’ and ‘responsive’ gene signatures in our own single-cell RNAseq dataset (Data ref: Netskar et al, 2024) comprising peripheral blood, tissue-derived and tumor-infiltrating NK cells (427 donors, seven different solid tumors), we observed a strong overlap between ‘anergic’ and CD56^bright^ NK cells (Fig. 1C). We hypothesize that the increased cytotoxic potential observed in Egr2 siRNA-treated anergic NK cells could be a consequence of deleting a transcription factor linked to the CD56^bright^ state, rather than an ‘exhausted’ state. Further phenotypic characterization of the NK cells before and after Egr2 downregulation would be valuable for shedding light on this matter. Hence, the relationship between the anergic signature found in tumor-infiltrating NK cells and the well-documented transcription-independent hypofunctionality of NK cells lacking self-specific inhibitory receptors requires further study. Understanding how these anergic states develop and are maintained in the TME could assist in designing precise therapeutic interventions in the future.

Nevertheless, the findings of Sabag et al (2024) highlight the potential of targeting intracellular programs that govern NK cell differentiation and function through gene editing to enhance the functional template of NK cells for effective and durable immunotherapies. Future research should focus on refining these gene editing techniques and exploring their applicability to a broader range of immune cell dysfunctions. While the nanoparticle delivery system shows great promise, its efficacy and safety in diverse clinical settings requires further investigation.

In conclusion, the ability to reprogram anergic NK cells through targeted gene editing represents a step forward toward cancer immunotherapy. This approach holds the promise of improving the efficacy of cell-based therapies and ultimately achieving better outcomes for cancer patients.
